# Supplementary material for: Detailed molecular and epigenetic characterization of the pig IPEC-J2 and chicken SL-29 cell lines
Source: iScience. 2023 Feb 20;26(3):106252. doi: 10.1016/j.isci.2023.106252 (PMC10018572; doi:10.1016/j.isci.2023.106252)
Supplement: Data S1. Complete homer output for identified motifs in Pig IPECJ-2, related to Table 2 — Homer motif analysis results for histone modifications H3K4me1, H3K4me3, H3K27ac, and enhancer elements of pig IPECJ2 cell line. P-values >1e-10 are possible false positives. Within each folder (e.g. peak_files_CTCF) are the html files showing the identified motifs when using homer (e.g. homerResults.html). [file mmc2.zip › S5/Pig_IPECJ_2/peak_files_H3K4me3/homerResults/motif3.similar.html]

motif3

## Information for motif3

A
G
C
T
T
C
G
A
G
A
T
C
A
C
G
T
A
G
C
T
C
G
T
A
G
A
T
C
G
T
A
C
  
Reverse Opposite:  

C
A
T
G
C
T
A
G
G
C
A
T
C
T
G
A
T
G
C
A
C
T
A
G
A
G
C
T
T
C
G
A
  

|  |  |
| --- | --- |
| p-value: | 1e-220 |
| log p-value: | -5.067e+02 |
| Information Content per bp: | 1.619 |
| Number of Target Sequences with motif | 15143.0 |
| Percentage of Target Sequences with motif | 68.60% |
| Number of Background Sequences with motif | 15411.2 |
| Percentage of Background Sequences with motif | 58.24% |
| Average Position of motif in Targets | 149.1 +/- 84.8bp |
| Average Position of motif in Background | 150.4 +/- 94.4bp |
| Strand Bias (log2 ratio + to - strand density) | -0.0 |
| Multiplicity (# of sites on avg that occur together) | 1.49 |
| Motif File: | file (matrix) reverse opposite |

### Similar de novo motifs found

|  |  |  |  |  |  |  |  |
| --- | --- | --- | --- | --- | --- | --- | --- |
| Rank | Match Score | Redundant Motif | P-value | log P-value | % of Targets | % of Background | Motif file |
| 1 | 0.616 | G T C A A C G T C G A T T G C A G T A C T A G C A C T G T A C G | 1e-202 | -466.996352 | 45.88% | 35.90% | motif file (matrix) |
| 2 | 0.738 | G T C A T A G C A C G T G A C T G C T A A T G C A G T C T C A G T A C G A T G C | 1e-131 | -302.614946 | 34.56% | 27.07% | motif file (matrix) |
| 3 | 0.637 | A T C G T A C G T G C A C A T G A T C G C A G T C T G A C T G A | 1e-127 | -292.601985 | 33.69% | 26.38% | motif file (matrix) |
| 4 | 0.682 | C G T A C G T A A C T G A C T G A C G T C G T A C G T A A T C G A G T C A C T G | 1e-113 | -262.027229 | 30.00% | 23.34% | motif file (matrix) |
| 5 | 0.723 | G T A C A C T G C T A G T C A G C G A T A C T G G T C A A T C G C G A T T C G A | 1e-69 | -160.742560 | 4.34% | 2.33% | motif file (matrix) |
| 6 | 0.623 | T A G C T A G C G T A C T C A G C A T G C G T A G A T C C G A T G T A C C G T A G T A C G A C T T C G A | 1e-50 | -115.861085 | 15.15% | 11.78% | motif file (matrix) |
| 7 | 0.740 | C A T G T A G C C T A G C A G T C G T A G A T C G A C T G A C T C G T A T A G C | 1e-23 | -53.147943 | 0.54% | 0.19% | motif file (matrix) |
